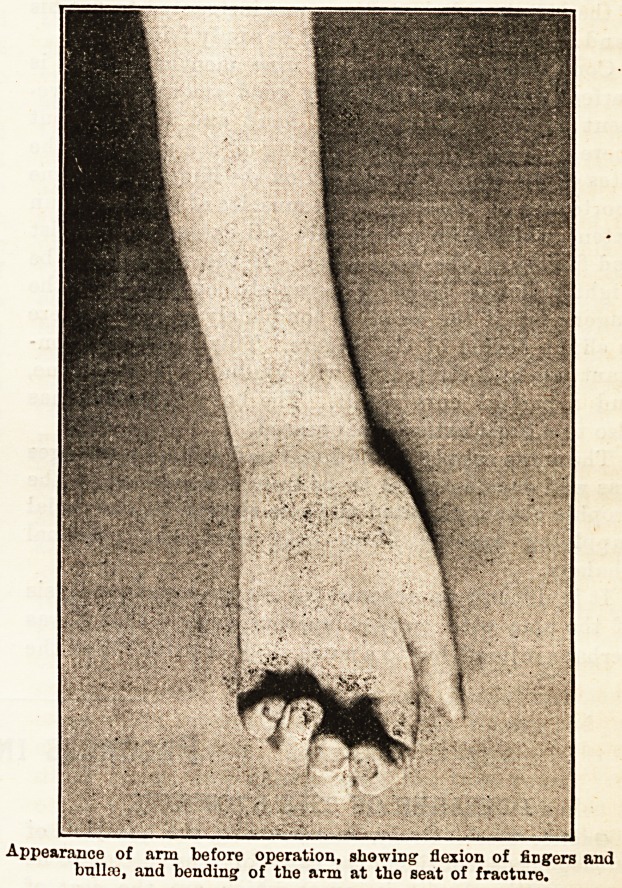# A Case of Fracture of Rad and Ulna, with Injury to the Median Nerve

**Published:** 1895-09-28

**Authors:** Wheelton Hind

**Affiliations:** Assistant-Surgeon to the North Staffordshire Infirmary


### MEDICAL PROGRESS AND HOSPITAL CLINICS.

[*The Editor will be glad to receive offers of co-operation and contributions from members of the profession. All letters should be addressed to* THE EDITOR, THE LODGE, PORCHESTER SQUARE, LONDON, W.]

### A CASE OF FRACTURE OF THE RADIUS AND ULNA, WITH INJURY TO THE MEDIAN NERVE.—CONSEQUENT PARALYSIS AND ATROPHIC LESIONS.—OPERATION.—RECOVERY.

By WHEELTON HIND, M.D., Assistant-Surgeon to the North Staffordshire Infirmary.

Late in March of this year I was consulted by Mr. S——, who told me that on December 1st, 1888, while attempting to arrange some strapping on machinery in motion, his left arm became caught, and was dragged into the machinery. The result was a fracture of both bones. Medical aid was obtained and splints applied, and the usual line of treatment was adopted for some weeks. When, however, the splints were removed, the forearm and hand were useless, and there was some loss of sensation in the parts supplied by the palmar branches of the median nerve. There was a good deal of callus, and though there was good bony union, the bones were not in good position, and there was a good deal of thickening in the muscles of the forearm, and another thickening just above the ends of the radius and ulna. About the end of March, finding that the state of things was gradually becoming worse, he came into my hands.

Condition: There were signs of recent fracture. The bones had united at a slight angle, the fracture having been about the centre of the forearm. There was also some thickening just above the wrist.

The fingers were extended as far as the proximal phalanges, the more distant ones being flexed. The thumb was extended. The skin was cold, blue, and moist with perspiration, and raised into large bullæ on the radial sides of thumb, index, and middle fingers, as far as the junction of the middle and proximal phalanges. There was loss of sensation over the whole of the median side of the palm of the hand and the palmar surface of the fingers. Sensation was impaired in the ring and little fingers, but not absent. There was great atrophy of the muscles of the palm, especially those of the thenar eminence, also of the interossei, slight atrophy of the muscles of the hypothenar eminence. There was absolute loss of movement of thumb, and of all power of flexion of the fingers, except to a slight degree in the little finger; pronation was almost lost.

The vesicular bullæ were stated to be only a few days old. I judged from the symptoms that both the ulnar and median nerves had been implicated, but the ulnar to a much smaller degree, and thinking that, probably, the nerves were pressed upon, or involved in callus, I determined to cut down and free them. The first point to decide was which of the two swellings was the more likely to be the seat of the injury to the nerve; there being no symptom, however, to point more certainly to one rather than the other, it was resolved to seek for the nerve at the point of fracture. Mr. Folker and Mr. Alcock kindly assisted me, chloroform being administered by Dr. McAldonie. A median incision was made in the arm for about 2½ inches, and the fascia divided. I exposed the ulna nerve from this situation, but it seemed perfectly healthy. Next I sought the median, but it was not in its normal situation, and after some dissecting and searching deep down in the arm, I prolonged the incision downwards, towards the wrist, where I exposed the distal part of the nerve in its normal situation. It was then an easy matter to trace it upwards. It dipped downwards beneath the deep layer of muscles, and lay in contact with the radius, to which it was very intimately adherent. Here the nerve was very considerably atrophied and changed in texture for about three-quarters of an inch, it being less than half the size of the nerve below. It was carefully dissected away

Appearance of arm before operation, showing flexion of fingers and bullæ, and bending of the arm at the seat of fracture.

from the bone, and a small rough corner of callus removed by bone forceps, and the bones were forcibly bent, in order to straighten them.

The nerve was slung to its proper position by a loop of catgut, to prevent subsequent adhesion to the bone, which was brought out of the edges of the wound, and a portion of muscular tissue thrust under it. Antiseptic dressings were applied, and the arm was put on a splint. The bandages had to be slackened in the first twenty-four hours on account of slight swelling. The first dressing was on April 3rd—the third day. The gut ligature holding the nerve in position was removed. There was considerable tension in the wound, and a good deal of serous discharge from the drainage tube; but it was pleasing to note that sensation had returned all over the hand. The wound was redressed in two days, when it was looking more healthy, and the skin of the palm of hand was dry and more healthy in texture; the sores on the thumb and fingers were healing rapidly, and granulating well. On April 10th a small slough of tendon or fascia came away, and all stitches were removed. The wound was doing well, and the fingers healing, sensation becoming more acute, and there were some slight and tremulous movements in the palm of the hand; two days later there was considerable movement of thumb, but one or two superficial stitch abscesses gave trouble. The sore on the index finger was not doing so well as the others. From this date improvement was rapid, and the splint was left off four weeks after the operation.

Forced movement was then begun; this was followed by some slight contraction of the muscles of the forearm, which was treated by occasional re-application of the splint, and the patient was instructed to use his hand as much as possible, and to apply friction.

Condition at the end of nine months: There is perfect sensation, and a very good amount of movement in the muscles of the hand and forearm, but there still remains some glazing of the skin over the sites of the trophic ulceration on the fingers, and some shortening of the long flexor muscles of the arm to an extent which does not permit full extension of wrist and fingers at the same time. If the wrist joint be slightly flexed there is complete extension of the fingers, but if the wrist be completely extended there is slight flexion of the fingers. This I hope by constant use and stretching will gradually be overcome, and a perfect cure result. The bony deformity has also to a great extent disappeared.

The nerve supply to the distal and middle phalanges was well demonstrated to be from the median by the trophic changes limited to these situations, the radial supplying sensory fibres only over the proximal phalanx.

It is difficult to account for the apparent paralysis of the ulna with marked wasting, except that it was perhaps injured, but to a much slighter extent than the median. I think the atrophy of the muscles it supplied was too well marked to be due to disuse only, the arm having been splinted only for about six weeks and then being taken down to allow the muscles to be used.

No doubt the present shortening of the flexor muscles is due to rupture and injury at the time of the accident; there was much recent repair to be seen in them during the dissection for the operation.

The report after seven years is that the nutrition of the fingers is perfect, and that the same thing may be said about movement and position.

Only a few months ago another case of injury to the median nerve came under my notice. The patient was a groom who had cut his wrist one inch above the annular ligament in the left hand. The cicatrix was painful and swollen, and beneath it a hardened mass the size of a kidney bean could be felt. There was ulceration and sclerosis of the skin at the top of the middle and first fingers, and the growth of the nail was irregular. Sensation also impaired. Some months after the accident I was asked to see him, and I advised operation.

On cutting down I found the median nerve almost severed, and the upper divided end bulbous. Dissecting up to the annular ligament, I fastened the proximal and distal ends and stitched them together with fine silk ligatures, and closed the wound. The distal part of the nerve was so atrophied that I was doubtful whether it would regain its power. But the case did well. In a few days the wounds healed on the finger, sensation began to return, and the man has been able to return to his work.

In both cases the chief complaint was the trophic change in the fingers, the paralysis being to a great extent neglected on the part of the patient, owing to the fact that the fingers being sore the loss of power was not brought to notice.

Wounds of nerves are a very satisfactory class of injuries to deal with, and if possible it should be made certain at the time whether a nerve has been involved in a wound or no, but even after an interval of months or years operative measures should not be given up as hopeless.

## Figures and Tables

**Figure f1:**